# Hyaluronic acid treatment outcome on the post-extraction wound healing in patients with poorly controlled type 2 diabetes: A randomized controlled split-mouth study

**DOI:** 10.4317/medoral.23061

**Published:** 2020-02-10

**Authors:** Saša Marin, Snježana Popović-Pejičić, Bojana Radošević-Carić, Nataša Trtić, Zoran Tatić, Srećko Selaković

**Affiliations:** 1 Oral Surgery Specialist, PhD, DDM. Department of Oral Surgery, Faculty of Medicine, University of Banja Luka, Bosnia and Herzegovina; 2 Full Professor, PhD, DDM. Department of Endocrinology, Faculty of Medicine, University of Banja Luka, Bosnia and Herzegovina; 3 Assistant Professor, PhD, DDM. Department of Endocrinology, Faculty of Medicine, University of Banja Luka, Bosnia and Herzegovina; 4 Assistant Professor, PhD, DDS. Department of Periodontology and Oral Health, Faculty of Medicine, University of Banja Luka, Bosnia and Herzegovina; 5 Associate Professor, PhD, DDS. Department of Oral Surgery, Faculty of Medicine, University of Banja Luka, Bosnia and Herzegovina; 6 Clinic of Dental Medicine, Military Medical Academy, Belgrade, Serbia.; 7 Full Professor, PhD, DDS. Dental Clinic of Vojvodina, Medical Faculty, University of Novi Sad, Serbia

## Abstract

**Background:**

Hyaluronic acid is widely used in the medical field. However, there is a lack of research about its effect on patients with certain risks, such as compromised wound healing commonly found in patients with poorly controlled type 2 diabetes. The aim of this study is to investigate the efficacy of hyaluronic acid on the post-extraction wound healing and pain in patients with poorly controlled type 2 diabetes.

**Material and Methods:**

The randomized controlled split-mouth study was designed, which included 30 patients with poorly controlled type 2 diabetes with a bilaterally same teeth in the lower jaw for extraction. The sockets treated with 0.8% hyaluronic acid represented the study group, while the sockets where hyaluronic acid was not applied represented the control group. Wound closure rate (WCR), clinical scores in wound healing scale (WHS) and pain intensity in Visual analogue scale (VAS) were recorded. Patients were followed up on 5th, 10th, 15th, 20th, 25th day after tooth extraction.

**Results:**

The results showed a higher WCR at the extraction site where hyaluronic acid was applied. Also, statistically significant difference was found (*p*< 0.001). In regards to WHS, the sockets treated with hyaluronic acid showed better healing, especially on day 10 (*p*=0.006) and day 15 (*p*=0.021). However, there were no statistically significant differences in VAS scores between groups.

**Conclusions:**

Hyaluronic acid placed in post-extraction socket in patients with poorly controlled diabetes may improve wound healing, especially in the first days after application.

** Key words:**Hyaluronic acid, type 2 diabetes mellitus, post-extraction wound healing, wound closure measurement.

## Introduction

Type 2 diabetes mellitus is a chronic metabolic disorder, and its prevalence has been increasing globally (1). The chronic hyperglycemia increases risk of microvascular and macrovascular complications associated with diabetes (2,3). Consequently, microcirculatory deficiencies and impaired leukocyte function can impact the wound healing processes, such as tissue nutrition, inflammatory response, and tissue permeability (4,5). As a result, poorly controlled diabetes is associated with a series of complications and unsatisfying treatment outcomes.

Intraorally, patients with poorly controlled diabetes could be expected to suffer similar complications and be susceptible to oral diseases and dental problems (6,7). This is evident in the healing of post-extraction wounds, which is often delayed and followed with unfavourable post-extraction socket changes (8,9). Due to the bone alteration, patients with poorly controlled diabetes suffer from impaired osseointegration, elevated risk of peri-implantitis, and higher level of implant failure (10).

Hyaluronic acid (HA) is a high-molecular glycosaminoglycan which can be found as a constituent of the connective tissue, synovial fluid, skin, and other body tissues (11). Previous studies have reported that HA performs a great number of functions. For instance, it exerts an anti-inflammatory effect during oral wound healing, supports the integrity of tissues regarding osmotic pressure and tissue lubrication, and the viscosity of joint synovial fluid (12-14).

Owing to biocompatible features and participation in biological processes related to tissue healing, HA has become widely used in dentistry (15). The studies have demonstrated the beneficial role of HA on swelling, pain and anti-inflammatory efficiency in oral surgery (16,17). Furthermore, some authors reported its role in bone repair by facilitating cell's migration, proliferation and differentiation (18).

Despite the fact that HA is widely used in the medical field, there is a lack of research about clinical applications of HA and its effects on patients with certain risks, such as compromised wound healing commonly found in patients with poorly controlled type 2 diabetes.

The aim of this study was to investigate the influence of HA on the wound healing after tooth extraction for patients with poorly controlled type 2 diabetes.

- Abbreviations in text

HA - Hyaluronic acid; HbA1c - Glycated haemoglobin; WCR - Wound closure rate;

WHS - Wound healing scale; VAS - Visual analogue scale.

## Material and Methods

After obtaining written approval from the local ethics committee (01-9-331.2/13) and signed informed consent from each patient, this study was conducted at the Department of Oral Surgery, Faculty of Medicine, University of Banja Luka and at the Department of Endocrinology, Faculty of Medicine, University of Banja Luka between May 2014 and September 2018.

**Figure 1:**
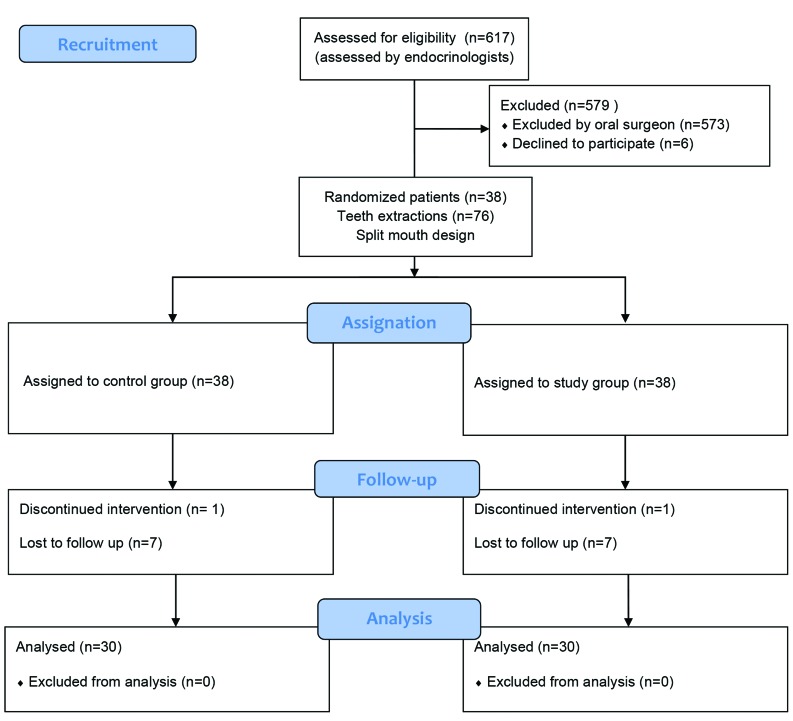
Flow chart of the study.

In order to conduct this research and address the research objectives, the authors carried out a randomized controlled split-mouth study among patients who required extraction of two same bilaterally anterior teeth in the lower mouth jaw (Fig. 1). Hence, this ensured that the same patient had a control site and a study site. The sockets treated with HA represented study group. The sockets where HA was not applied served as a control group, and the wound healed naturally by clot formation. Randomization and group allocation was performed prior to extractions for each patient using a computer software (Research Randomizer, Geoffrey C. Urbaniak & Scott Plous, USA).

Inclusion criteria for selecting patients for the study were: 1) confirmation of type 2 diabetes with glycated haemoglobin (HbA1c) range 8% - 10% (63.9 mmol/l - 86 mmol/l); 2) confirmation of at least one vascular complications of diabetes (except acute coronary syndrome and stroke); 3) patients older than 18 years undergoing bilaterally anterior teeth extraction in the lower jaw without acute infection presence (prosthetic reasons, advanced tooth decay, failed endodontic treatment and periodontal reasons) ; 4) minimally invasive extraction or extraction with minimal trauma. Exclusion criteria included: 1) patients with type 2 diabetes mellitus with HbA1C < 8% (63.9 mmol/l) and >10% (86 mmol/l); 2) patients with complications of diabetes such as acute coronary syndrome and stroke six months prior to teeth extraction; 3) patients with a history of allergies or adverse effects to local anaesthetics; 4) patients who used antibiotics and anti-inflammatory medication within two weeks prior to teeth extraction; 5) patients under corticosteroids treatment; 6) patients with bleeding problems and acute infection; and 7) patients who use tobacco.

All tooth extractions were performed by the same oral surgeon while all measurements were conducted by a blinded operator (a colleague outside of the research study, who measured and tracked clinical healing process). Extractions were done under local anaesthesia 3% Mepivacaine Hydrochloride (Scandonest 3% plain, Septodont, Saint-Maur-des-Fossés, France). After tooth extracting at the study site, 0.8% hyaluronic acid (Gengigel Prof, Ricefarma srl, Milan, Italy) was applied in the socket, while at the control site nothing was applied. The patients were instructed not to eat or drink one hour after HA treatment. Once patients were included in the study, wound closure rate (WCR), scores in wound healing scale (WHS) and pain intensity were recorded.

- Variables

**Figure 2:**
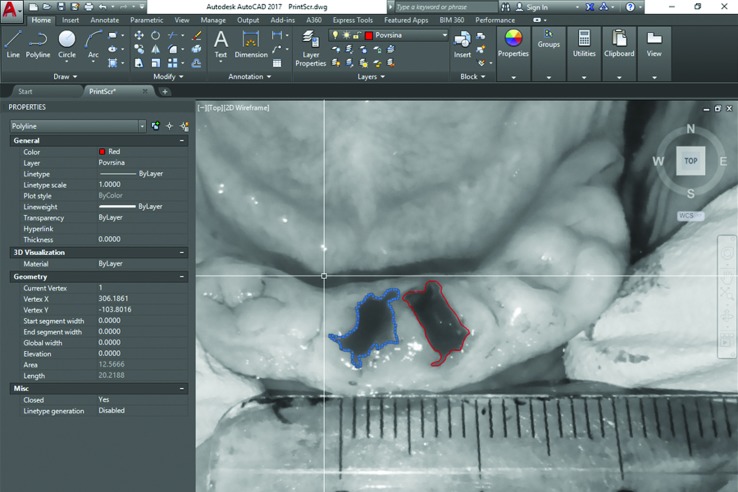
Using Autocad programme (Autodesk, Mill Valley, California, United States) to calculate wound surface in square millimetres. Blue color represents study group; Red color represents control group.

The wound area was evaluated using digital photographic controls with a ruler (Nikon D5100, Nikon Corporation, Tokyo, Japan) from fixed heights and Autocad (computer-aided-drafting (CAD)) (Autodesk, Mill Valley, California, United States) programme. In Autocad, after setting one milimetre value with benchmark, polylines in the form of closed polygons overlaid the wound area and wound surface measurements were automatically obtained in square millimetres (Fig. 2).

Following this, WCR was expressed as a percentage with the initial value of the first day wound healing rate marked as “0%”. The subsequent measurements were taken during recalls on 5th, 10th, 15th, 20th, 25th day, and compared with the initial value obtained on first day.

Clinical healing was investigated using a modified WHS by Vandana Shenoy *et al*., developed to evaluate post-extraction sockets healing (19). Wound healing was categorised into excellent (no signs of inflammation of surrounding tissue, good amount of epithelium, completely filled socket with granulation tissue), good (mild to no inflammation of surrounding soft tissue, good amount of epithelium, socket was completely filled with granulation tissue), moderate (moderate inflammation of soft tissue, moderate amount of epithelium, half to two-thirds of socket was filled with granulation tissue), and poor (moderate or severe inflammation of surrounding soft tissue, exposed bone surface and insufficient amount of granulation tissue). Patients were followed up on 5th, 10th, 15th, 20th, 25th day after tooth extraction.

Pain intensity was recorded on the 1st, 2nd and 3rd day after the extraction using Visual analogue scale (VAS). According to the scale, 0 indicated "absence of pain" and 10 indicated "excessive pain". Patients were instructed how to use VAS scale during first three days after intervention.

- Statistical methods

The statistical analysis was performed using the SPSS 21.0 package programme (IBM SPSS statistics, IBM Corporation, New York, United States). The statistical significance of differences among goups were assessed using ANOVA for repeated measures for WCR and VAS. For the comparison between control and study group, with respect to clinical WHS, Fisher’s exact test was used. The *p* value of less than 0.05 for the 95% confidence interval was accepted as significant.

## Results

Six hundred and seventeen patients were screened and 38 patients fulfilled the inclusion criteria. This split-mouth study included 30 patients (53.33% males; 46.67% females; mean age 59.5±9.37). A total of eight patients were excluded from the study since seven patients did not come on all recall dates. In the case of one patient, a tooth was fractured during extraction and *p* minimally invasive procedure could not be performed. The glycated haemoglobin was in range 8.1% - 9.5%.

The results showed an increased WCR among groups for each subsequent recall. At the first visit following tooth extraction (5th day), WCR for the control group was 29.11% and for the HA group 51.35% while at their last visit (25th day) WCR for the control group was 74.53% and for the HA group 84.36%. Statistically significant difference among groups was found (*p*< 0.001, F=22.057, df=1). More details are presented in Table 1.

The comparison of both groups with respect to clinical WHS was done and the statistically significant differences were found between groups on day 10 (*p*=0.006) and day 15 (*p*=0.021). There were no significant differences on day 5 (*p*=0.069), day 20 (*p*=0.308) and day 25 (*p*=0.521). On the last day of follow-up, 63.33% excellent healing score was found in the control group and 76.67% in the HA group. During recalls on day 20 and day 25, no poor healing was found in both groups (Table 2).

**Table 1:**
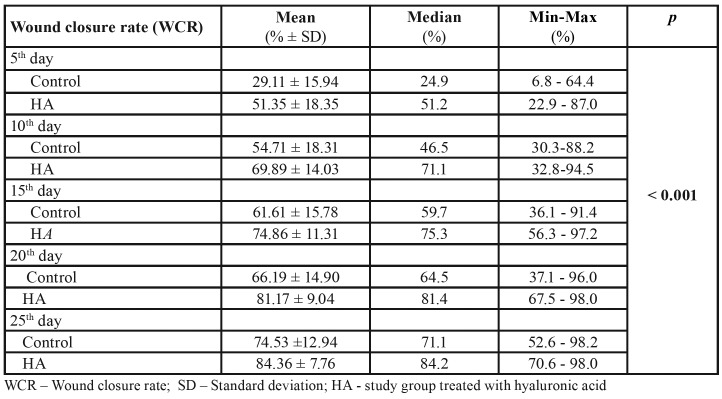
Wound closure rate compared between the control group and study group (HA).

**Table 2:**
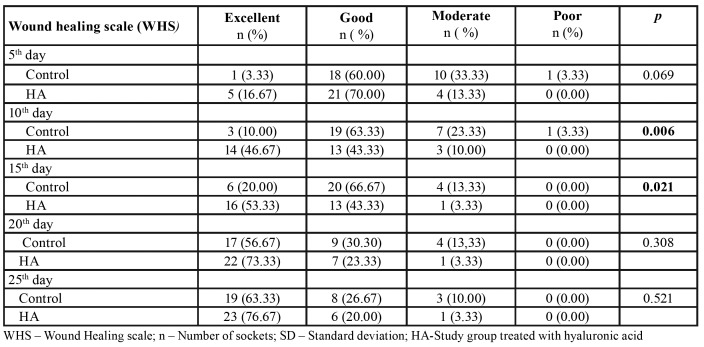
Clinical wound healing compared between the control group and study group (HA).

Pain scores on VAS decreased during follow-up period; for the control group from 2.68 to 0.36 and for the HA group from 3.04 to 0.60. The statistically significant difference among groups during follow-up period was not found (*p*=0.324, F=0.989, df=1) (Table 3).

**Table 3:**
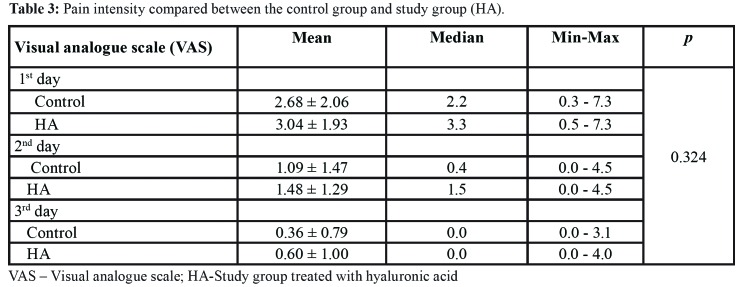
Pain intensity compared between the control group and study group (HA).

Discussion 

The study focused on the efficiency of HA in healing process after tooth extractions at patients with poorly controlled type 2 diabetes. There are few studies that investigated the role of HA in wound healing process. These previous studies found that numerous properties are important for wound healing, such as promotion of epithelization and granulation tissue formation (20-22). On the other hand, there is little information on the effectiveness of HA for treatments of patients with poorly controlled diabetes.

Regarding the wound closure measurements, a statistically significant difference was found. At the extraction site where HA was applied, results showed a higher WCR. The largest discrepancy between groups was found on the fifth day, decreasing in the next post-extraction days, while on day 25th it was the lowest.

These findings accord with scores in WHS, which showed statistical significance between groups only on day 10 (*p*=0.006) and day 15 (*p*=0.021). These results may be attributed to the ability of HA to reduce the penetration of bacteria into the tissue by creating barrier. Hyaluronic acid retains the water in aqueous solution and maintains the stiffness that acts as a antimictobal barrier, directly after HA application (15,23,24). Gocmen *et al*. confirmed that HA has an anti-inflammatory effect and that it improves angiogenesis after one week following extraction (17). These results agree with our findings in which the significant effect of HA on wound healing was observed in the early period following tooth extraction. Our findings differ from the results showed by Galli *et al*. (25). The authors investigated whether HA appears to improve wound healing over surgical incision. Scoring by each clinician, they did not find a significant difference between placebo and HA group. Healing of tooth extraction socket in patients with poorly controlled diabetes is often delayed due to the duration of hyperglycemia, impaired recruitment of inflammatory cells and microcirculation (26). It is interesting to note that poor healing score and delayed wound healing was reported only in the control group on up to day 10 (3.33%). No cases of poor healing and no significant differences were found between groups on day 20 (*p*=0.308) and 25 (*p*=0.521). Although different reasons for teeth extractions in patients with poorly controlled diabetes might affect the wound healing, it should be noted that the risk of poor healing was decreased with minimally invasive extractions and the exclusion of cases with presence of acute infections, severe oral conditions and tooth fractures.

One of the aims in many studies is to reduce postoperative pain, and to find an alternative anlagetic option with minimal adverse effects. In cases after minimal invasive tooth extraction, the pain intensity usually subsides in the first three days after intervention. Several studies reported that HA reduces pain by anti-inflammatory and anti-oedematous contribution (27). Due to its positive effect and abovementioned features, HA has been widely used in medical practice (28,29). In our study, we did not find any correlation between the application of HA and decreasing pain after teeth extraction. Our study is in line with Gocmen *et al*. study in which no significant differences were found in pain intensity according to VAS score although less inflammatory responses occurred in cases where HA was applied (17). Korey et el. reported that HA has a role in swelling reduction and less alveolar osteitis occurrence after third molar extraction but they did not find its contribution in decreasing pain measured with VAS (16). Contrary to our results, Yilmaz *et al*. demonstrated that local administration of HA into extraction sockets decreased the pain after the procedure (30).

It should be mentioned that many different methods for wound healing measurement have been reported in literature (31-33). For this study, digital photograph assessment was employed since it is currently widely used in practice. To the best of our knowledge, this is the first study to evaluate the healings of oral wounds by using digital photograph assessment and Autocad programme. Limitations of this study mainly concern the number of patients and the performance of minimally invasive extractions. Also, in this split-mouth study design some wounds were located close to each other. It could increase possibility of wound contamination from one socket to another during healing process. Additional research studies with a larger sample size of patients with poorly controlled diabetes are needed to confirm our findings.

## Conclusions

Based on our results, 0.8% hyaluronic acid placed in post-extraction socket in patients with poorly controlled diabetes may improve wound healing, especially in the first days after application. Evaluating the effect of HA after more invasive or surgical extractions in patients with compromised wound healing would be worth analysing.
